# Editorial: Emerging talents in pharmacology: Drugs outcomes research and policies 2022

**DOI:** 10.3389/fphar.2023.1162703

**Published:** 2023-03-02

**Authors:** Rachel M. A. Linger, Joseph O. Fadare, Ye Shen, Lon J. Van Winkle

**Affiliations:** ^1^ Department of Biomedical Sciences, Rocky Vista University, Parker, CO, United States; ^2^ Department of Pharmacology and Therapeutics, College of Medicine, Ekiti State University, Ado-Ekiti, Nigeria; ^3^ Department of Epidemiology and Biostatistics, College of Public Health, University of Georgia, Athens, GA, United States; ^4^ Department of Biochemistry, Midwestern University, Downers Grove, IL, United States; ^5^ Department of Medical Humanities, Rocky Vista University, Parker, CO, United States

**Keywords:** drugs, outcomes, policies, therapies, medication, health, pharmacology, students

## Introduction

This Research Topic is a contribution to the “emerging talents” article Research Topic dedicated to highlighting the work of student researchers. It is focused on the field of drug outcomes research and policies.

Across the world, students are undertaking key research as part of their education in Drug Outcomes Research and Policies; however, most of this research is not communicated to the wider audience. We recognize that this is because many student researchers find the thought of peer-review daunting. At Frontiers, peer-review is considered a collaborative process and the interactive peer-review is tailored to provide hands-on guidance and constructive feedback to researchers. Frontiers is committed to the development of emerging talents and wants to see student researchers strive for success at publications.

The research presented here highlights the quality and diversity of student researchers across the field of Drug Outcomes Research and Policies. For instance, one coauthor is working toward a Ph.D. in June 2024 because he feels that clinical practice should pay more attention to polypharmacy and potentially inappropriate medication use. The coauthor of another paper completed a Ph.D. in Pharmaceutical Sciences and Pharmacogenomics and is now working toward completion of post-doctoral research of real-world data science in June of this year. Dr. Radtke transitioned from a focus on rare diseases as an undergraduate to infectious diseases and global health, because these diseases are among the top causes of preventable child deaths worldwide. Unlike the findings presented in other papers in this series, less data are often available for the effectiveness of drugs in children especially for infectious diseases. And when these data are available, they are frequently underutilized (Radtke and Butte, 2022).

## Improving drug treatment outcomes

As expected from the preceding perspective, most studies in this Research Topic concerned adults. Aspirin is an inexpensive antiplatelet agent with a long history of widespread use for secondary prevention of atherosclerotic cardiovascular disease (ASCVD) events and mortality. Within the last 10 years, mounting evidence has questioned the benefit provided by low-dose aspirin (75–325 mg daily) in primary prevention of ASCVD events. In 2022, a systematic review demonstrated a small but significant reduction in cardiovascular disease events (Guirguis-Blake et al., 2022). This evidence supports updated recommendations from the United States Prevention Services Task Force on the use of aspirin for the primary prevention of cardiovascular disease (US Preventive Services Task Force, 2022). An important unanswered question is how to identify patients that will benefit most from aspirin treatment in the setting of primary prevention. Hu et al. conducted a meta-analysis of studies measuring changes in carotid intima-media thickness (cIMT) as a biomarker for asymptomatic carotid atherosclerosis. While aspirin treatment reduced progression of cIMT in a subset analysis, no differences in cardiovascular disease events or mortality were observed. The benefits of aspirin treatment for secondary prevention of atherosclerotic cardiovascular disease events and mortality are fortunate indeed, but unfortunately this inexpensive treatment offers no such advantage for patients with asymptomatic carotid artery atherosclerosis.

Like cardiovascular disease, diabetes is a global health problem with significant economic and quality of life impact on patients worldwide. Treatment often involves expensive medications that must be self-injected subcutaneously. As new products become available, it is important to consider not only safety and efficacy, but also the economic impact for patients and healthcare systems. Han et al. conducted a cost-utility analysis of IDegLira in China, which became available in that country in April 2022. IDegLira is a fixed-dose combination of insulin degludec and the glucagon-like peptide 1 receptor agonist, liraglutide. While treatment of type 2 diabetes mellitus using IDegLira is more convenient for patients and likely to be cost-effective in several countries (Davies et al., 2016; Ericsson and Lundqvist, 2017; Hunt et al., 2017; Psota et al., 2017; Raya et al., 2019), the analysis conducted by Han et al. indicates that monotherapy with either drug alone is more cost effective in the Chinese national healthcare system. These data will be informative for policymakers as they navigate decisions related to IDegLira use in China.

One example of government regulated drug access is the National Centralized Drug Procurement (NCDP) in China. Huang et al. investigated the knowledge, attitudes and practice of pharmacists and other healthcare professionals towards NCDP, which is aimed at reducing drug costs for patients and healthcare systems. While over 40% of those surveyed had good knowledge about NCDP, less than one-quarter had positive attitudes and good practices. A primary factor contributing to negative attitudes and poor practices was lack of confidence in the safety and efficacy of generic NCDP drugs compared to the brand-name products. This study suggests that more robust clinical evidence to support the equivalence of NCDP generics and brand-name drugs coupled with training of pharmacists and healthcare professionals may ultimately reduce drug costs.

Similarly, available drugs might be used more appropriately through interventions with healthcare providers. Tian et al. investigated the impact of the expert consensus on safety management of polypharmacy issued by the Chinese Association of Geriatric Research. In the study of outpatient prescriptions from tertiary hospitals in Chengdu, a city in southwestern China’s Sichuan province, the expert guidance initially curbed both polypharmacy and potentially inappropriate medication use. However, the effect was lost over time and the authors infer that a multi-pronged approach may produce more sustainable effects.

Along with provider training and policy implementation, the success of patient treatment can be improved through development of new therapies. The paper by Wu et al. shows how the use of bicarbonate Ringer’s solution after liver transplant better protects against acute kidney injury than more commonly used crystalloid solutions, while Jenkins et al. review current as well as developing therapies to lower urate levels and, thus, help prevent/treat gout.

## Conclusion

Articles in this Research Topic present the work of student researchers including a perspective on evidence-based pediatric therapies, reviews of urate-lowering therapies and aspirin, two investigations of policy impact, a cost-utility analysis of a novel combination drug product, and a clinical trial of a novel crystalloid solution. Together, this Research Topic of articles highlights the diverse contributions of trainee researchers to drug outcomes research and policy around the world.

## References

[B1] Davies M. J. Glah D. Chubb B. Konidaris G. Mcewan P. (2016). Cost effectiveness of IDegLira vs. Alternative basal insulin intensification therapies in patients with type 2 diabetes mellitus uncontrolled on basal insulin in a UK setting. Pharmacoeconomics 34, 953–966. 10.1007/s40273-016-0433-9 27438706

[B2] Ericsson A. Lundqvist A. (2017). Cost effectiveness of insulin degludec plus liraglutide (IDegLira) in a fixed combination for uncontrolled type 2 diabetes mellitus in Sweden. Appl. Health Econ. Health Policy 15, 237–248. 10.1007/s40258-016-0301-y 28063135 PMC5343072

[B3] Guirguis-BlakeJ. M.EvansC. V.PerdueL. A.BeanS. I.SengerC. A. (2022). Aspirin use to prevent cardiovascular disease and colorectal cancer: Updated evidence report and systematic review for the US preventive Services Task Force. JAMA 327 (16), 1585–1597. 10.1001/jama.2022.3337 35471507

[B4] Hunt B. Mocarski M. Valentine W. J. Langer J. (2017). Evaluation of the long-term cost-effectiveness of IDegLira versus liraglutide added to basal insulin for patients with type 2 diabetes failing to achieve glycemic control on basal insulin in the USA. J. Med. Econ. 20, 663–670. 10.1080/13696998.2017.1301943 28294641

[B5] Psota M. Psenkova M. B. Racekova N. Ramirez De Arellano A. Vandebrouck T. Hunt B. (2017). Cost-effectiveness analysis of IDegLira versus basal-bolus insulin for patients with type 2 diabetes in the Slovak health system. Clin. Outcomes Res. 9, 749–762. 10.2147/CEOR.S143127 PMC5731336 29276398

[B6] Raya P. M. Blasco F. J. A. Hunt B. Martin V. Thorsted B. L. Basse A. (2019). Evaluating the long-term cost-effectiveness of fixed-ratio combination insulin degludec/liraglutide (IDegLira) for type 2 diabetes in Spain based on real-world clinical evidence. Diabetes Obes. Metab. 21, 1349–1356. 10.1111/dom.13660 30740861 PMC6594226

[B7] US Preventive Services Task Force Davidson K. W. Barry M. J. Mangione C. M. Cabana M. Chelmow D. Coker T. R. (2022). Aspirin use to prevent cardiovascular disease: US preventive Services Task Force recommendation statement. JAMA. 327 (16), 1577–1584. 10.1001/jama.2022.4983 35471505

